# RadA, a MSCRAMM Adhesin of the Dominant Symbiote *Ruminococcus gnavus* E1, Binds Human Immunoglobulins and Intestinal Mucins

**DOI:** 10.3390/biom11111613

**Published:** 2021-10-31

**Authors:** Marc Maresca, Radia Alatou, Ange Pujol, Cendrine Nicoletti, Josette Perrier, Thierry Giardina, Gwenola Simon, Vincent Méjean, Michel Fons

**Affiliations:** 1 Aix Marseille University, CNRS, Centrale Marseille, ISM2, IM2B, 13007 Marseille, France; angepujol@hotmail.com (A.P.); cendrine.nicoletti@univ-amu.fr (C.N.); josette.perrier@univ-amu.fr (J.P.); thierry.giardina@univ-amu.fr (T.G.); 2 Laboratoire de Biologie Moléculaire et Cellulaire, Université des Frères Mentouri Constantine 1, RN79 Constantine, Algeria; alatouradia@yahoo.com; 3 Aix Marseille University, Université de Toulon, CNRS, IRD, MIO UM 110, 13007 Marseille, France; gwenola.simon@univ-amu.fr; 4 Aix Marseille University, CNRS, BIP UMR7281, IMM, IM2B, 13007 Marseille, France; mejean@imm.cnrs.fr

**Keywords:** *Ruminococcus gnavus*, adhesin, bacterial Ig-like domain, collagen, mucus, mucin, solid phase assay, Caco-2, HT-29-16E

## Abstract

Adhesion to the digestive mucosa is considered a key factor for bacterial persistence within the gut. In this study, we show that *Ruminococcus gnavus* E1 can express the *radA* gene, which encodes an adhesin of the MSCRAMMs family, only when it colonizes the gut. The RadA N-terminal region contains an all-β bacterial Ig-like domain known to interact with collagens. We observed that it preferentially binds human immunoglobulins (IgA and IgG) and intestinal mucins. Using deglycosylated substrates, we also showed that the RadA N-terminal region recognizes two different types of motifs, the protein backbone of human IgG and the glycan structure of mucins. Finally, competition assays with lectins and free monosaccharides identified Galactose and N-Acetyl-Galactosamine motifs as specific targets for the binding of RadA to mucins and the surface of human epithelial cells.

## 1. Introduction

Adhesion is a general strategy developed by microorganisms to colonize complex ecosystems. In the digestive tract, adhesion has often been considered a prerequisite for bacteria to stably persist. In their pioneer work, Gordon and co-workers illustrated the crosstalk between the host and the resident microbiota, using *Bacteroides thetaiotaomicron* as a model bacterium [1,2]. It was then shown that different bacteria are able to exchange information with the host, impacting the expression of various eukaryotic genes. Today, adhesion can be considered not only a colonization factor but also an element that allows communication between the host and the microbiota. Both pathogenic and commensal bacteria have developed various adhesive structures and mechanisms to bind to the targets they are susceptible to encounter in the host (reviewed by Pizarro-Cerdá and Cossart [3], and Stones and Krachler [4]).

In Gram-positive bacteria, the family of MSCRAMMs (Microbial Surface Components Recognizing Adhesive Matrix Molecules) proteins shares the following common features. MSCRAMMs are exported proteins anchored to the bacterial cell wall mainly by family 1 sortase enzymes, and they mediate the adhesion of the bacterium to host surfaces [5].

Many bacterial pathogens exhibit MSCRAMMs, which bind to various extracellular matrix proteins such as collagens. The paradigm of the MSCRAMMs family is the Cna protein of *Staphylococcus aureus* [6]. MSCRAMMs of the CNA-like family of collagen-binding adhesins are structurally related and are found in many Gram-positive bacterial species such as Ace in *Enterococcus faecalis* [7], Acm in *Enterococcus faecium* [8] or Cnm in *Streptococcus mutans* [9]. They constitute virulence factors in different models of infectious diseases and mediate bacterial adhesion to collagen-rich tissues [10].

For symbiotic bacteria of the gut microbiota, mucus that covers the intestinal epithelium is the preferred target for adhesion. Mucus is a complex mixture of membrane-bound and secreted glycoproteins where mucins are the major structural components [11]. Mucus plays a dual role: it constitutes a physical and biochemical barrier protecting the epithelial monolayer and, in addition, is a source of energy for the gut microbiota [12,13,14]. In humans, mucins are encoded by more than 20 different genes. They are mainly constituted by an O-linked N-acetylgalactosamine (GalNAc) glycoprotein core structure that is further elongated and often modified [15,16,17].

Different MSCRAMMs that target mucus have been described in lactobacilli [18,19]. However, little is known concerning the MSCRAMMs of gut-dominant strict anaerobic bacteria. *Ruminococcus gnavus* is one of the predominant species of the digestive tract retrieved in 90% of humans [20]. The *R. gnavus* strain E1 was isolated from the dominant fecal microbiota of a healthy volunteer [21]. We previously showed that crosstalk exists between the E1 strain and the host. For example, the expression of the bacteriocin encoding clusters *rumA* and *rumC* depends upon the proteolytic activity of trypsin [22,23], which is a gut-associated characteristic [24]. Moreover, trypsin is also involved in the maturation of RumC [25,26]. In addition, the E1 strain induces the expression of different genes encoding glycosyl transferases and mucins [27]. The aim of this work was to characterize a microbial adhesin of the E1 strain that we called RadA. RadA displays typical features of the MSCRAMMs adhesins family and may be involved in better communication between this bacterium and its host. Here, we show that the N-terminal domain of RadA, although interacting with collagens, such as other known MSCRAMMs, preferentially recognizes human immunoglobulins (IgG and IgA) and the intestinal mucins.

## 2. Results

### 2.1. radA Cluster Analysis

**Figure 1:**
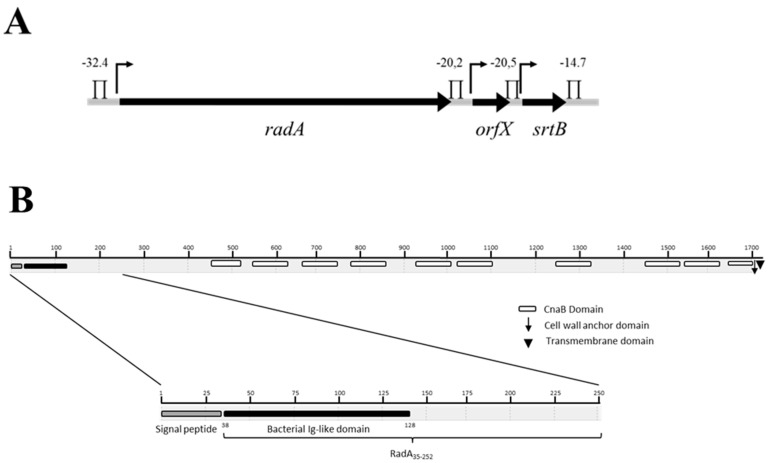
Schematic representation of the *radA* cluster (**A**) and of the RadA domains (**B**)**.** In Figure 1A, *radA*, *orfX* and *srtB* coding sequences are represented as black arrows and intergenic regions as grey lines. *radA*, *orfX* and *srtB length* (5310 bp, 630 bp and 669 bp, respectively) is not scaled. ↱ stands for putative promotors. ∏ indicates putative Rho-independent transcription terminators and the corresponding theoretical ΔG_0_ expressed as kCal/mol. In Figure 1B, □ represents the CnaB domains, ↓ the LPQTP cell wall anchor domain, and ▼ the C-terminal transmembrane domain.

In previous work, we showed that the *R. gnavus* strain E1 harbors two genetic clusters, respectively, involved in the biosynthesis of two bacteriocins: RumA [22] and RumC [28]. When the chromosomal region (7.5 kb long) located immediately upstream of the *rumA* cluster was analyzed, three contiguous genes were identified and were subsequently called *radA*, *orfX* and *srtB* (Figure 1A).

*radA*, *orfX* and *srtB* start codons were preceded by potential −30 and −10 regions and ribosome binding sites. The intergenic regions contained possible rho-independent terminators, suggesting that the three genes constituted independent transcriptional units.

OrfX is 630 bp long and could encode a conserved protein of unknown function exhibiting similarities with previously predicted proteins from *Clostridium nexile* DSM 1787 or *Clostridium hathewayi* DSM13479. *srtB* is 669 bp long and codes for a putative protein exhibiting 41% identity with StrB sortases of *Coprococcus comes* ATCC27758 and *Dorea formicigenerens* ATCC27755. *radA* codes for a 1769 amino acids protein that exhibits strong homologies with different adhesion proteins homologous to the Cna-type adhesin of *Staphylococcus aureus* [29] that belongs to the MSCRAMMs family [30]. More than 90% identity was obtained with putative proteins from *Eubacterium ramulus* ATCC29099 (Sequence ID: gb|ERK52358.1|) and *Tyzzerella nexilis* DSM 1787 (gb|EEA8230{Citation}7.1|) reference genomes for the Human Microbiome Project. Characteristic structural motifs were found all over the deduced RadA-primary structure (Figure 1B). A signal peptide was predicted from residues 1 to 32, suggesting that RadA is exported. An all-β bacterial Ig-like domain (from residues 38 to 128) was identified, probably able to interact with a wide variety of extracellular matrix components. Ten CnaB-like domains (from residues 447 to 512, 554 to 624, 668 to 747, 789 to 867, 903 to 1007, 1044 to 1105, 1227 to 1313, 1343 to 1422, 1452 to 1517 and 1630 to 1706) were predicted by Pfam analysis. Finally, an unusual putative cell-wall anchor domain (LPQTP, compared to the LPXTG consensual motif) and a transmembrane domain (at the end of the protein) were identified. Interestingly, the putative adhesin, which was detected in the DSM1787, strain harbored the very same LPQTP cell wall anchor domain. Moreover, synteny analysis revealed that the same organization of these three genes is detected in *C. nexile* DSM1787.

### 2.2. The radA Gene Is Only Expressed “In Situ”

**Figure 2:**
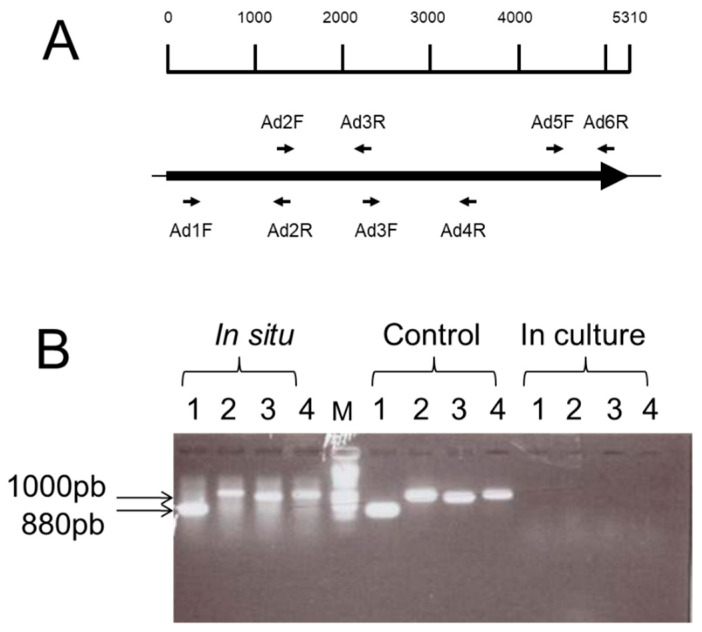
Schematic localization of the PCR-primers along the *radA* gene (**A**) and expression of *radA* (**B**). *In situ*: RT-PCR on total RNA extracted from E1-mono-contaminated rats. In culture: RT-PCR on total RNA extracted from E1 culture in BHI-YH medium. Control: PCR on E1 chromosome DNA. Lines 1, 2, 3 and 4 correspond to PCR reactions carried out with couples of primers Ad5F-Ad6R, Ad3F-Ad4R, Ad2F-Ad3R and Ad1F-Ad2R, respectively. M stands for the GeneRuler 1kb DNA Ladder marker. 1000 bp and 800 bp with arrows point to the size of the products.

The expression of the *radA* gene was assessed in various cells growth conditions, either in rich BHI-YH medium or when *R. gnavus* E1 colonized the digestive tract of gnotobiotic rats. Reverse transcription and PCR amplification were carried out on total RNA purified from the *caecal* content of *R. gnavus* E1-monocontaminated animals, using converging primers of specific regions of the *radA* gene (Figure 2A). Similar experiments were carried out on total RNA obtained from late exponential-phase cultures of *R. gnavus* E1 in a liquid culture medium. Strikingly, the analysis of the PCR products showed that the *radA* gene was expressed in situ when *R. gnavus* E1 colonized the digestive tract of the animals, whereas it was below detection level when the E1 strain was grown until the late exponential phase in the liquid culture medium (Figure 2B).

### 2.3. Heterologous Expression of GST-RadA_35-252_

**Figure 3:**
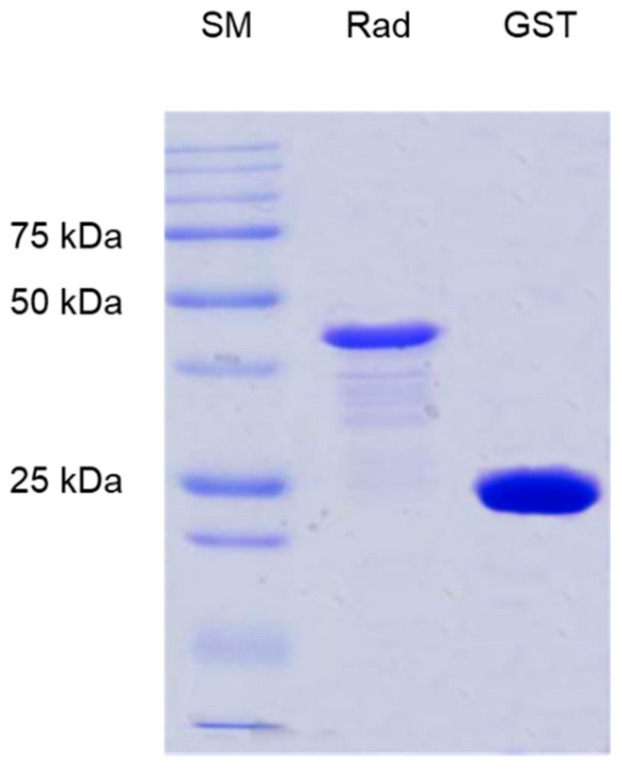
Analysis of purified GST-RadA_35-252_, by SDS-PAGE. SM, standard marker; Rad, GST-RadA_35-252_; GST, glutathione S-transferase; 25 kDa, 50 kDa and 75 kDa indicate the molecular weight of three proteins present in the SM.

Previous work reported that the region involved in the recognition of extracellular matrix components by CNA-like adhesins was located in the N-terminal part of the protein, upstream from the repeated domains (Zong et al., 2005). In particular, all-β bacterial Ig-like domains have been previously reported to interact with a wide variety of extracellular matrix components. We thus decided to explore the corresponding region of RadA (Figure 1B). In order to identify the potential partners recognized by this domain, the DNA fragment encoding the N-terminal region from Leu_35_ to Leu_252_ residues was cloned in the pGEX4T1 vector and heterologously expressed in the *E. coli* BL21 strain as an N-terminal GST-tagged fusion protein. After purification, one band appeared on SDS-PAGE with an apparent molecular mass of 48 kDa (Figure 3)**,** slightly lower than the theoretical value (51.982 kDa).

The band was purified, digested with endolysin-C, and the resulting peptides were subjected to Edman degradation. The peptides match the GST-RadA fragments and correspond to the C-terminus part of GST fused to the N-terminus part of RadA that is devoid of its putative signal sequence. Thus, we confirmed that the purified protein corresponded to the expected hybrid protein, i.e., to GST fused to the N-terminus part of RadA devoid of its putative signal sequence. This hybrid protein was called GST-RadA_35-252_ and contained the Ig-like domain from residues 38 to 128.

### 2.4. GST-RadA_35-252_ Binds to Immunoglobulins and Mucins

**Figure 4:**
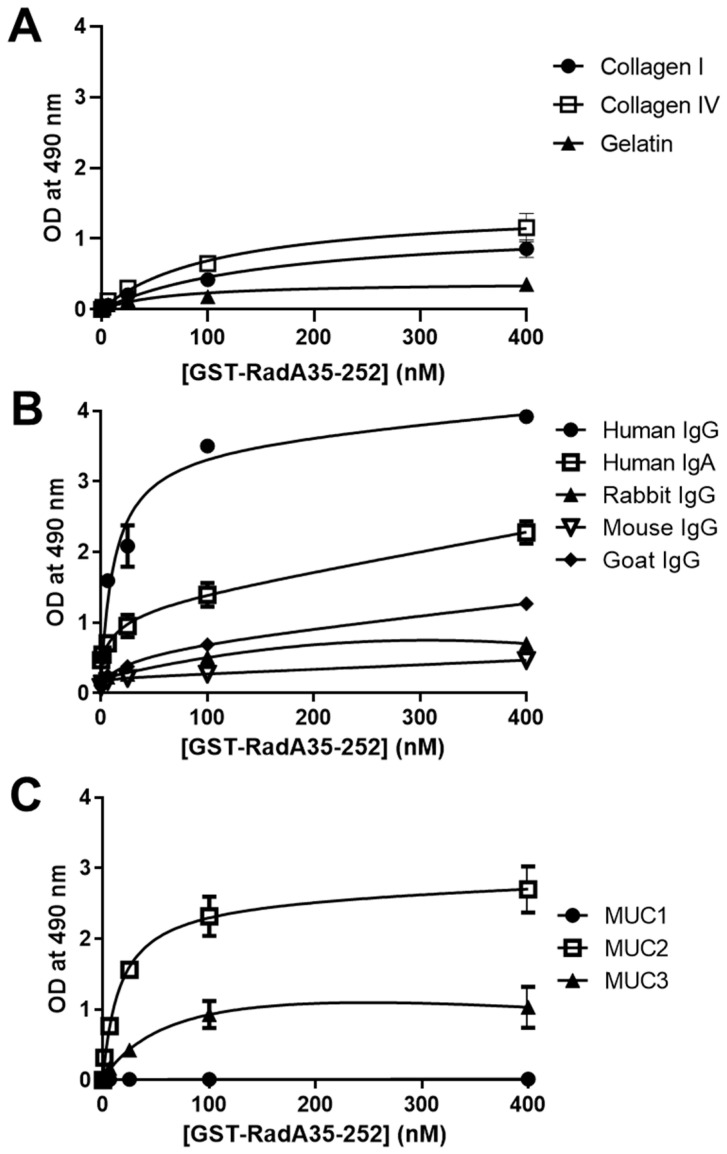
GST-RadA_35-252_ binds preferentially to human immunoglobulins and mucins. The interaction of GST-RadA_35-252_ with collagen I, collagen IV or gelatin (**A**) immunoglobins from various animal species (**B**) or with mucins type I (MUC1), type II (MUC2) or type III (MUC3) (**C**) was studied by the solid-phase binding assay as described in Material and Methods. All tested proteins were coated at 4 pmol per wells (40 nM). Results are expressed as means ± S.D. (*n* = 3).

As *R. gnavus* is one of the dominant species present in the digestive tract of 90% of humans [20], in addition to collagens, the capability of the RadA Ig-like domain to adhere to the main components encountered in mucus, i.e., mucins and immunoglobulins, was tested. Preliminary experiments performed with pure GST used as a negative control showed that pure GST did not show any binding with any of the substrates tested. The interaction of the RadA Ig-like domain (GST-RadA_35-252_) with different coated proteins was carried out by solid-phase assay (Figure 4, Table 1). Gelatin was used as negative control and showed a low ability to interact with GST-RadA_35-252_ with a binding capacity (Bmax) of 0.38 ± 0.13 and an affinity constant (Km) of 66.18 ± 18.49 nM (Figure 4A). Collagens I and IV were then tested based on the homology of GST-RadA_35-252_ with other known CNA-like proteins (Figure 4A). As expected, results confirmed the ability of GST-RadA_35-252_ to interact weakly with collagens with Bmax values of 0.82 ± 0.11 and 1.16 ± 0.14 and Km values of 161.9 ± 23.81 and 112.5 ± 21.86 nM for collagen I and collagen IV, respectively (Table 1). Regarding immunoglobulins, the interaction of GST-RadA_35-252_ was the highest towards IgG from humans (i.e., Bmax of 3.92 ± 0.47 and Km of 13.75 ± 0.34), compared to IgG from other animal species (Figure 4B, Table 1). Although weaker, results showed a significant interaction of GST-RadA_35-252_ with human IgA (i.e., Bmax of 2.14 ± 0.46 and Km of 24.71 ± 1.86 nM). Finally, the interaction of GST-RadA_35-252_ with intestinal mucins (type I (MUC1), type II (MUC2) and type III (MUC3)) was evaluated (Figure 4C) [31]. The highest binding capacity of GST-RadA_35-253_ was observed towards the type II mucin (Bmax of 2.56 ± 0.23 and Km of 16.61 ± 0.46 nM) followed by the type III mucin (Bmax of 1.06 ± 0.38 and Km of 78.52 ± 6.43 nM) (Table 1).

Almost no interaction was observed with the membrane-associated mucin type I with a Bmax of 0.08 ± 0.02 and a Km of 110.1 ± 30.12 nM. Overall, the solid-phase binding assay showed that although GST-RadA_35-252_ interacts with collagens I and IV, in accordance with its homology with other CNA-like proteins, it preferentially binds to human immunoglobulins (IgA and IgG) and intestinal mucins (mucins type II and III) (Table 1).

### 2.5. GST- RadA_35-252_ Recognizes a Carbohydrate and a Peptide Motif

**Figure 5:**
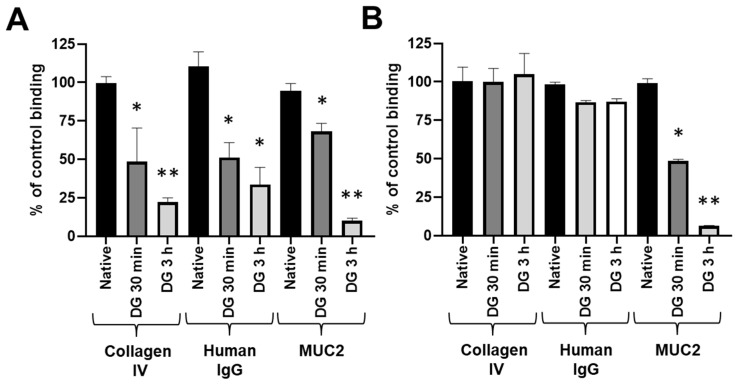
The interaction of GST-RadA_35-252_ with MUC2 depends on a glucidic motif. The binding of WGA (**A**) or GST-RadA_35-252_ (**B**) to native and deglycosylated (DG) collagen IV, human IgG or mucin type II (MUC2) (coated at 4 pmol per wells (40 nM)) was measured as explained in Materials and Methods. Results are expressed as means ± S.D. with *: *p* < 0.05; **: *p* < 0.01 (*n* = 3).

Having shown that the RadA Ig-like domain interacts at the same time with collagens, immunoglobulins and mucins, the role of the carbohydrate motifs exhibited by these proteins and recognized by GST-RadA_35-252_ was evaluated using collagen IV, human IgG and mucin type II as model proteins. Collagen IV, human IgG and mucin type II were chemically deglycosylated for 30 min (i.e., DG 30 min) or 3 h (i.e., DG 3 h), and their interaction with GST-RadA_35-252_ was then evaluated. Efficient deglycosylation of each substrate was confirmed using a solid-phase binding assay with the lectin wheat-germ agglutinin (Figure 5A).

**Figure 6:**
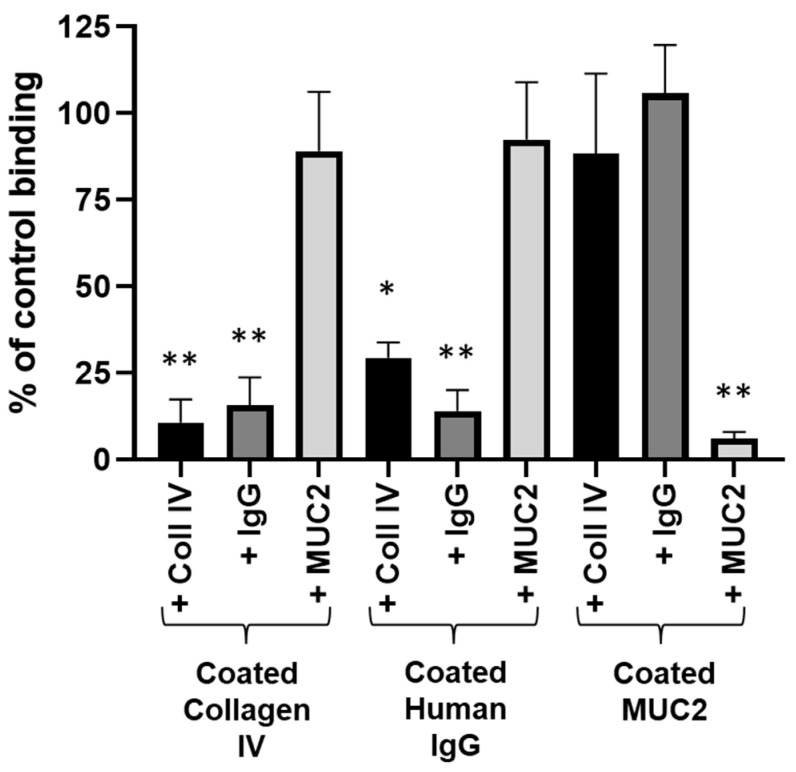
The interaction of GST-RadA_35-252_ with collagen IV, human IgG or type II mucin involves two separate domains/mechanisms. GST-RadA_35-252_ was pre-incubated with soluble collagen IV, human IgG or type II mucin (MUC2) (400 nM) before being added to wells coated with collagen IV, human IgG or type II mucin (MUC2) (protein-coated at 4 pmol per wells (40 nM)) as explained in Materials and Methods. Results are expressed as means ± S.D. with *: *p* < 0.01, **: *p* < 0.001 (*n* = 3).

Interestingly, deglycosylation had a dramatic effect on GST-RadA_35-252_ adhesion to type II mucin (51.43 ± 1.05 and 93.62 ± 0.28% inhibition of the binding of GST-RadA_35-252_ to type II mucin DG 30 min and DG 3 h, respectively), whereas it had no significant effect on adhesion of GST-RadA_35-252_ to collagen IV or human IgG (Figure 5B). These results strongly suggest that the N-terminal part of RadA binds two types of substrates: a carbohydrate moiety for type II mucin and a peptide backbone for collagen IV and human IgG. To test this hypothesis, the GST-RadA_35-252_ was pre-incubated in the presence of free collagen IV, human IgG or type II mucin, and its capability to bind to wells coated with those substrates was then checked. In this assay, pre-incubation of GST-RadA_35-252_ with collagen IV or human IgG led to 70–90% inhibition of its adhesion to the corresponding coated substrate (coated collagen IV or human IgG) but did not significantly affect the binding capacity of GST-RadA_35-252_ to coated type II mucin (Figure 6).

Similarly, pre-incubation of GST-RadA_35-252_ with type II mucin did not significantly modify further adhesion to coated collagen IV or human IgG but led to 94.05 ± 2.99% inhibition of its adhesion to coated type II mucin (Figure 6). Taken together, these results demonstrated that although collagen IV and human IgG are in competition for the binding to GST-RadA_35-252_, they do not cross-compete with type II mucin. This suggests that they do not interact with the same adhesion region of GST-RadA_35-252_. The interaction of GST-RadA_35-252_ with collagen IV and human IgG is furthermore independent of their glucidic moiety, whereas its interaction with type II mucin depends on a carbohydrate motif.

### 2.6. GST-RadA_35-252_ Recognizes a Specific Carbohydrate Motif of Type II Mucin

**Figure 7:**
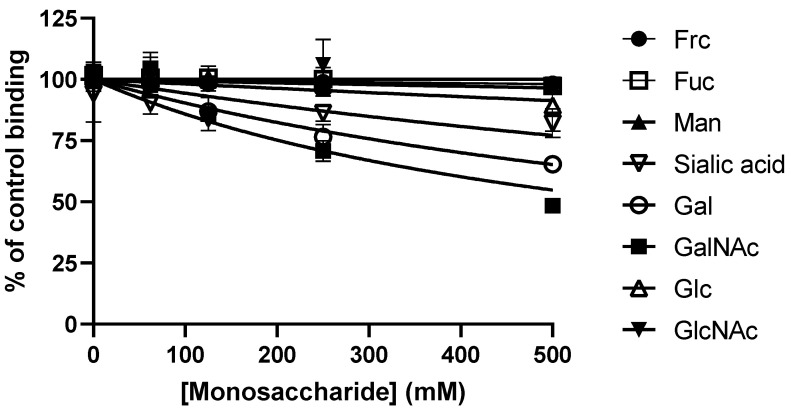
Galactose and N-acetyl-Galactosamine in solution inhibit the interaction of GST-RadA_35-252_ with type II mucin. GST-RadA_35-252_ was incubated with monosaccharides in solution before being added to coated type II mucin (MUC2) (coated at 4 pmol per wells (40 nM)) as explained in Materials and Methods. Tested monosaccharides were Fructose (Frc), Fucose (Fuc), Mannose (Man), Sialic acid, Galactose (Gal), N-acetyl-galactosamine (GalNAc), Glucose (Glc) and N-acetyl-glucosamine (GlcNAc). Results are expressed as means ± S.D. (*n* = 3).

To identify the carbohydrate motif recognized by GST-RadA_35-252_ during the adhesion to type II mucin, competition experiments were carried out. GST-RadA_35-252_ was pre-incubated in the presence of increasing concentrations (from 0 to 500 mM) of various carbohydrates (Frc, Fuc, Gal, GalNAc, Glc, GlcNAc, Man or sialic acid) before it was used in binding assays on coated type II mucin (Figure 7).

Among the various carbohydrates tested, only Gal and GalNAc significantly decreased the adhesion of GST-RadA_35-252_ to type II mucin, causing 34.68 ± 2.73 and 51.61 ± 3.41% inhibition at 500 mM (IC_50_ values of 937.2 ± 173.3 and 506.1 ± 139.1 mM, respectively) (Table 2).

**Figure 8:**
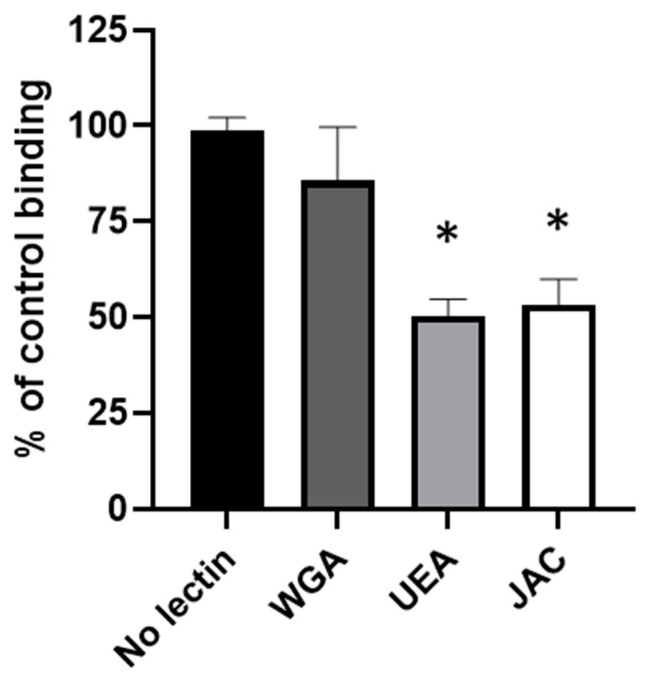
Lectins binding Galactose and N-acetyl-Galactosamine inhibit the interaction of GST-RadA_35-252_ with type II mucin. Coated type II mucin (coated at 4 pmol per wells (40 nM)) was pre-incubated with various lectins: Wheat Germ Agglutinin (WGA, specific for GlcNAc), Ulex Europaeus Agglutinin (UEA, specific of Gal) and Jacalin (JAC, specific of Gal and GalNAc) (all at 100 µg/mL). A solid-phase binding assay of GST-RadA_35-252_ was then conducted as explained in Materials and Methods. Results are expressed as means ± S.D. *: *p* < 0.01 (*n* = 3).

The involvement of Gal and GalNAc in the interaction of GST-RadA_35-252_ with type II mucin was furthermore confirmed by a competitive binding assay using lectins (Figure 8). Tested lectins were Wheat Germ Agglutinin (WGA) specific to GlcNAc, Ulex Europaeus Agglutinin (UEA) specific to Gal and Jacalin (JAC) specific to Gal and GalNAc. Among the three tested lectins, only lectins recognizing Gal and/or GalNAc (i.e., UEA and JAC) caused a significant inhibition of the binding of GST-RadA_35-252_ (i.e., 14.21 ± 13.89, 49.60 ± 4.38 and 46.67 ± 6.57% inhibition for WGA, UEA and JAC, respectively), confirming the role of these carbohydrates in the interaction of GST-RadA_35-252_ with type II mucin.

### 2.7. GST-RadA_35-252_ Binds to the Surface of Human Intestinal Epithelial Cells

**Figure 9:**
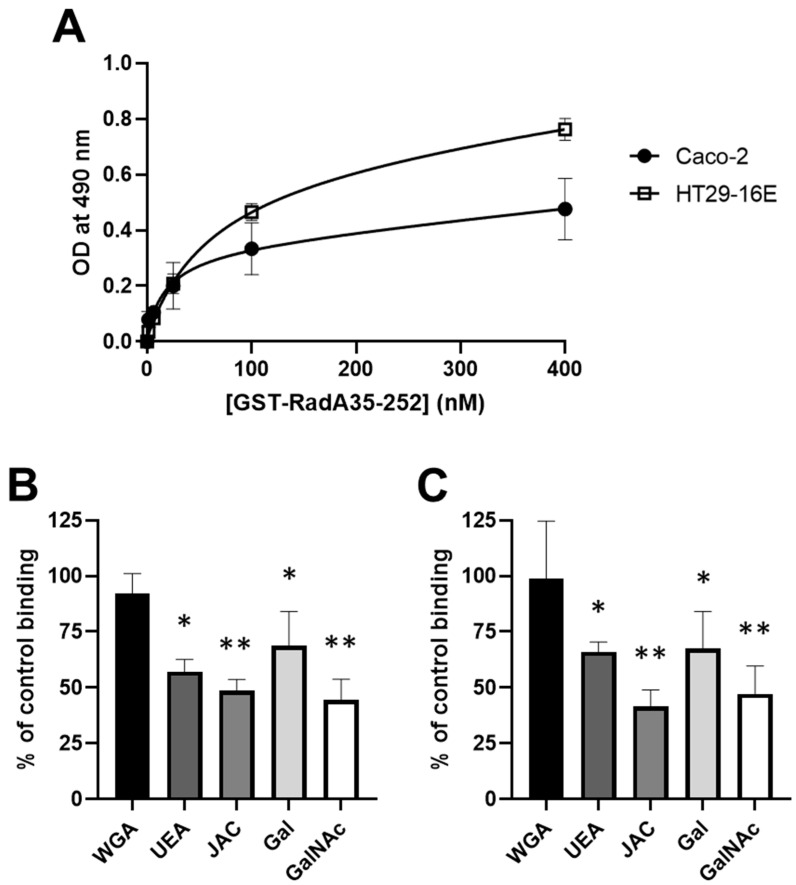
GST-RadA_35-252_ binds to the surface of human intestinal epithelial cells. Caco-2 and HT29-16E cells were used to study the dose-dependent binding of GST-RadA_35-252_ to human intestinal cells (**A**). The inhibitory effect of lectins and monosaccharides on the binding of RadA_35-252_ to Caco-2 cells (**B**) or HT29-16E cells (**C**) was studied as indicated in the Materials and Methods. Results are expressed as means ± S.D. *: *p* < 0.05, **: *p* < 0.01 (*n* = 3).

Based on the demonstrated interaction of GST-RadA_35-252_ with pure intestinal mucins in a solid-phase assay, its ability to bind to the mucins present on the surface of human intestinal cells was then evaluated using cell-based ELISA. Caco-2 and HT29-16E cells were used as models of human enterocyte and goblet cells, respectively (Ajandouz et al., 2016; Graziani et al., 2019). Binding parameters were first measured after the incubation of human intestinal epithelial cells with an increasing concentration of GST-RadA_35-252_ (Figure 9A). In accordance with the fact that HT29-16E cells secrete more mucins than Caco-2 cells, results showed that GST-RadA_35-252_ possesses a higher binding capacity to HT29-16E cells compared to Caco-2 cells (Bmax of 0.83 ± 0.17 and 0.49 ± 0.11 for HT29-16E and Caco-2 cells, respectively), the affinity constant of GST-RadA_35-252_ for Caco-2 cells being however lower than the one for HT29-16E cells (Km of 92.38 ± 15.21 and 34.83 ± 6.89 nM for HT29-16E and Caco-2 cells, respectively). To confirm the involvement of Gal and GalNAc in the interaction of GST-RadA_35-252_ with intestinal mucins, the effect of lectins (at 100 µg/mL) or Gal and GalNAc (at 500 mM) on the binding of GST-RadA_35-252_ to human intestinal cells was further evaluated (Figure 9B,C). Conversely, WGA did not cause significant inhibition of the binding of GST-RadA_35-252_ to the surface of human intestinal cells, pre-incubation of the human intestinal cells with UEA and JAC resulted in significant inhibition of GST-RadA_35-252_ binding (i.e., 45.37 ± 9.34 and 51.40 ± 5.12% or 34.07 ± 4.49 and 58.33 ± 7.34% inhibition for UEA and JAC on Caco-2 or HT29-16E cells, respectively). Similarly, pre-incubation of GST-RadA_35-252_ with Gal or GalNAc resulted in significant inhibition of its binding to Caco-2 and HT29-16E cells (i.e., 40.27 ± 5.92 and 47.56 ± 12.51% or 32.55 ± 16.72 and 52.88 ± 12.58% inhibition for Gal and GalNAc on Caco-2 or HT29-16E cells, respectively). The data obtained with human intestinal epithelial cells thus confirmed the results obtained with porcine intestinal mucins in a solid-phase binding assay and the role of Gal and GalNAc residues in the interaction of GST-RadA_35-252_ with intestinal mucins and epithelial cells.

## 3. Discussion

Digging into the genome sequence of *R. gnavus E1* revealed the presence of the *radA* gene coding for an adhesin belonging to the MSCRAMMs family. The bioinformatics analysis predicted the presence of a signal sequence at the N-terminal part of RadA, ten CnaB-like domains, and, at the C-terminus of the protein, a degenerate sortase-cleavage motif followed by a short hydrophobic segment and a final positively charged domain. These observations strongly suggest that RadA is exported and covalently linked to the cell wall by a sortase enzyme [5]. The presence of the *srtB* gene in the vicinity of *radA* agrees with this hypothesis. The sortase-cleavage motif we identified is LPQTP, which is different from the canonical LPXTG motif usually recognized by sortase A enzymes. Interestingly, the very same LPQTP motif is found in a putative adhesin of *C. nexile* strain DSM1787, which harbors a gene cluster similar to the *radA-orfX-srtB* locus of the E1 strain. Confirmation of the role of the product of *srtB* remains to be tested in the future. Previous works reported that the region involved in the recognition of extracellular matrix components by CNA-like adhesins is located in the N-terminal part of the protein, upstream of the repeated domains [29]. For the Cna adhesins of *S. aureus* and the AggLb adhesin of *Lactobacillus paracasei*, it was also proposed that the CnaB-domains act as a “stalk” that projects the N-terminal domain from the bacterial surface and facilitates its adherence to the matrix [32,33]. Similarly, we hypothesized that the 10 CnaB-like domains of RadA act as an antenna that projects the Ig-like domain and facilitates its adherence to mucus. We thus generated a GST-tagged fusion protein with the RadA region spanning from L_35_ to L_252_. The RadA_35-252_ fragment contains the predicted all-β bacterial Ig-like domain.

As *R. gnavus* E1 is a symbiotic bacterium of the human gut, we decided to evaluate the interaction of the RadA_35-252_ fragment not only with collagens but also with mucins and immunoglobulins present in the intestinal mucus. Although the RadA_35-252_ fragment interacts with collagens I and IV, in accordance with its homology with CNA-like protein, the most efficient binding capacity and affinity constants were observed with human immunoglobulins (IgA and IgG) and secreted intestinal mucins (type II and III mucins). Surprisingly, the results also showed that RadA_35-252_ does not bind to the membrane-associated type I mucin, and the reason for the specific interaction of RadA with type II and III mucins needs further investigation. The use of Caco-2 and HT29-16E cells (as models of enterocytes and goblet cells, respectively) allowed us to demonstrate that RadA_35-252_ not only binds to intestinal porcine mucins but also to the mucins expressed by human intestinal epithelial cells, strongly suggesting a potential role of RadA in the adhesion of *R. gnavus* E1 to the intestinal mucus and to the surface of the intestinal epithelium.

Using de-glycosylated substrates, we also showed that the RadA N-terminal region recognizes two different types of motifs, the protein backbone of collagens or human IgG and the glycan structure of type II mucin.

Competition assays carried out with lectins or free monosaccharides identified Gal and GalNAc as specific targets for the binding of RadA to mucins and the surface of human intestinal epithelial cells. It is noteworthy that *R. gnavus* E1 up-regulates the expression of different genes encoding glycosyl transferases and mucins by human intestinal goblet cells [27], providing further binding sites for E1 strain.

Furthermore, despite its inability to grow on mucins as a sole carbon source, previous studies identified 112 full-length genes encoding CAZymes (http://www.cazy.org) on the genome of *R. gnavus* E1 with many GH29 and GH95 fucosidases [34] and an overrepresentation of GH36 α-galactosidases [35,36,37]. Thus, it is tempting to hypothesize that RadA-mediated adhesion to mucus could play a crucial ecological role. It could provide *R. gnavus* E1 with a micro-environment where it is fed by other members of the microbiota that are able to break up complex carbohydrate chains issued from mucins in assimilable oligosaccharides. We propose that RadA helps to anchor *R. gnavus* E1 to the human mucus and intestinal epithelial cells, and we propose that RadA enhances the protective role of this strain, which produces the RumC bacteriocin in the gut ecosystem, activated by the human trypsin [23,25]. Finally, we showed that *radA* is expressed only when *R. gnavus* E1 colonizes the digestive tract (Figure 2). This observation suggests that the bacterium is able to sense gut-specific signals and that *radA* expression depends on dedicated regulators that remain to be identified.

## 4. Materials and Methods

### 4.1. Bacterial Strains and Media

The E1 strain was isolated from the predominant fecal microbiota of a healthy human adult [21] and further identified as *R. gnavus* [38]. It was grown in an anaerobic cabinet (Coy Laboratory Products, Grass Lake, MI., USA) in BHI-YH medium [22] at 37 °C. *Escherichia coli* BL21 (DE3) transformants were selected on LB medium containing 50 mg·L^−1^ ampicillin.

### 4.2. Animals, Ethics Statement and Experimental Design

Animal experiments were performed according to the guidelines of the French Ethics Committee, i.e., agreement N° A78-322-6. Six-week-old germ-free (GF) rats F344 from the ANAXEM platform (Micalis-INRAE, Jouy-en-Josas, France) were reared as previously described [23]. They were inoculated with around 10^9^ cells of the E1 strain (0.5 mL of fresh culture) by the intra-gastric route. After one week, individual fecal samples were collected and bacterial counts estimated. Then, the animals were sacrificed, and the cecal contents were collected.

### 4.3. Cloning

To explore the role of RadA, the N-terminus domain of the protein spanning from L_35_ to L_252_ residues was expressed as GST-tagged fusion protein, with the GST-Tag located at the N-terminus part of the chosen domain. The *radA* coding DNA region was PCR-amplified using DNA Taq-Polymerase HIFI (Invitrogen, Carlsbad, CA, USA) and specific primers Ol35-Eco and Ol252-Xho, allowing the addition of *Eco*RI and *Xho*I restriction sites at the 5′ and the 3′ ends of the fragments, respectively. (Detailed information concerning primers sequence and PCR conditions is given in Table 3.) The fragment was further cloned into the corresponding sites of the pGEX4T1 expression vector (Healthcare, Chicago, IL, USA). The recombinant plasmid was then introduced into *E. coli* BL21 (DE3) chemically competent cells.

### 4.4. Expression and Purification of Recombinant GST-Tagged Fusion Rad Domains

GST-tagged fusion protein (GST-RadA_35-252_) was expressed in *E. coli* BL21 (DE3) grown overnight at 17 °C in LB medium supplemented with ampicillin and IPTG (Sigma-Aldrich, Lyon, France). Bacterial lysis was carried out by incubating the cell pellet in “BugBuster Protein Extraction” reagent (Merck Millipore, Molsheim, France) (5 mL/g of cells) in the presence of benzonase nuclease (125 U/g) and lysozyme (200 mg/L) (Sigma-Aldrich) for 30 min at room temperature. The mixture was further centrifuged for 10 min at 10,000 *g*. GST-tagged fusion proteins present in the supernatant were purified with the GST.Bind^TM^ Kit (Novagen, Madison, WI, USA), according to the manufacturer’s instructions.

### 4.5. radA Expression

Isolated RNA obtained from mice caecal contents were further called “in situ RNA”. That obtained from E1 cultured cells was further called “In culture RNA”. Extraction of both types of RNA was carried out as described previously [28]. Four different regions of *radA* were PCR amplified after reverse transcription.

cDNA synthesis was performed by reverse transcription (RT) of 100 ng of “in situ-” or “in culture-RNA” primed with 50 ng of random hexamers (Invitrogen). The reaction was carried out at 50 °C for 1 h with the Superscript^TM^ III Reverse Transcriptase (Invitrogen) as recommended by the supplier. The enzyme was inactivated by heating for 15 min at 70 °C.

The analysis of the expression of the *radA* gene in situ was carried out directly using 1 µL of the “in situ” RT mixture as a template and 1 U of Platinium^Tm^ Taq DNA polymerase (Invitrogen). Similar reactions were carried out using 1 µL of the “in vivo” RT mixture as a template for analyzing the expression of the *radA* gene when *R. gnavus* E1 was grown in BHI-YH medium. Control reactions were also performed using an RT mixture without enzyme, an RT mixture without RNA or E1 chromosomal DNA as a template. (Detailed information concerning primers sequence and PCR conditions is given in Table 3.)

### 4.6. Bioinformatics Analysis

Manual validation of the automatic annotation was performed using the MicroScope platform [39]. Putative transcriptional terminators were predicted using the “RNA-fold program”. The sequences reported in this study were deposited in the ENA database under accession number LR882501.

### 4.7. Solid-Phase Binding Assays

The binding of GST-RadA_35-252_ to various proteins was determined using a solid-phase binding assay. Briefly, NUNC Maxisorp 96-well plates (Dominique Dutscher, Brumath, France) were coated overnight at 4 °C with 100 µL of tested proteins (final concentration: 40 nM, 4 pmol of protein per well) dissolved in coating buffer (50 mM sodium carbonate, pH 9.5). Tested proteins were gelatin (from pig), collagen I and IV (from mouse), IgA (from human), IgG (from human, goat, rabbit or mouse), type I mucin (from bovine submaxillary glands), type II mucin (from the porcine stomach) and type III mucin (from the porcine stomach) (Sigma-Aldrich, Lyon, France). The adsorption of each protein to the wells was confirmed using the BCA protein assay kit (Thermo Fisher Scientific, Illkirch-Graffenstaden, France). For each protein tested, more than 90% of the added protein was efficiently coated to the wells in these conditions. The next day, wells were washed three times with 230 µL of wash buffer (PBST: phosphate buffer saline (PBS) + 0.5% Tween 20 (*v*:*v*)). To block nonspecific binding, the wells were saturated for 1 h at 37 °C with 230 µL of 2% (*w*/*v*) gelatin dissolved in PBS. Gelatin was preferred to bovine serum albumin (BSA) since preliminary work showed that GST-RadA_35-252_ interacts strongly with BSA (data not shown). After three washes with PBST, wells were incubated at 37 °C for 2 h with increasing concentrations of GST-RadA_35-252_ (from 0 to 400 nM corresponding to 0 to 40 pmol per well) diluted in PBS + gelatin 2%. After three more washes, bound GST-RadA_35-252_ was detected by incubating wells for 1 h at 37 °C with rabbit anti-GST antibody (1:400 dilution) (Thermo Fisher Scientific). Pure GST (Merck, Fontenay-sous-Bois, France) was used as negative control and showed no interaction with any of the tested proteins (data not shown). Wells were next washed three times with PBST and incubated for 1 h at 37 °C with peroxidase-conjugated goat anti-rabbit antibody (1:10,000 dilution) (Jackson ImmunoResearch Laboratories, Cambridgeshire, United Kingdom). Wells were then washed six times with PBST and incubated with 100 µL of the peroxidase substrate o-phenylenediamine dihydrochloride (Sigma-Aldrich, Lyon, France) for 30 min at room temperature before the reaction was stopped by adding 50 µL of H_2_SO_4_. Finally, optical density (OD) at 490 nm was measured using a microplate reader (Biotek, Synergy Mx, Colmar, France). To evaluate the role of glycosylation in the interaction of GST-RadA_35-252_ with coated proteins, deglycosylated proteins (collagen IV, human IgG and type II mucin) were produced using a chemical deglycosylation kit following the manufacturer’s instructions (GlycoProfile^™^ IV Kit from Sigma-Aldrich). The GlycoProfile^™^ IV Kit utilizes trifluoromethanesulfonic acid (TFMS) in a deglycosylation system that completely removes all N- and O-linked glycans while preserving the protein/polypeptide structure. After deglycosylation, proteins were dialyzed using centrifugation onto Vivaspin^®^ 500, 10 kDa tubes (Sigma-Aldrich). The final protein concentrations were measured using a BCA protein assay (Thermo Fisher Scientific). Deglycosylated collagen IV, human IgG and MUC2 were then coated onto NUNC Maxisorb 96-well plates (final concentration: 40 nM, 4 pmol of protein per well) and subjected to solid-phase binding assay as explained above using GST-RadA_35-252_ at 100 nM. Competitive binding experiments were also conducted in which collagen IV, human IgG or type II mucin coated onto NUNC Maxisorp 96-well plates (final concentration: 40 nM, 4 pmol of protein per well) were exposed to GST-RadA_35-252_ (100 nM) pre-incubated or not for 30 min at 37 °C with soluble collagen IV, native human IgG or type II mucin (final concentration: 400 nM). Similarly, a solid-phase binding assay was performed using type II mucin coated onto NUNC Maxisorp 96-well plates (final concentration: 40 nM, 4 pmol of protein per well) and GST-RadA_35-252_ (100 nM) pre-incubated or not for 30 min at 37 °C with increasing concentration (from 0 to 500 mM, using 1:2 cascade dilution) of various monosaccharides: Fructose (Frc), Fucose (Fuc), Galactose (Gal), N-acetyl-galactosamine (GalNAc), Glucose (Glc), N-acetyl-glucosamine (GlcNAc), Mannose (Man) or sialic acid. Finally, a binding assay was also performed using type II mucin coated onto NUNC Maxisorp 96-well plates (final concentration: 40 nM, 4 pmol of protein per well) that were pre-incubated for 30 min at 37 °C with various lectins diluted at 100 µg/mL in PBS + gelatin 2%. Lectins tested were Wheat Germ Agglutinin (WGA), Ulex Europaeus Agglutinin (UEA) and Jacalin (JAC) (Vector Laboratories, Peterborough, United Kingdom). After three washes with PBST to remove unbound lectins, wells were incubated with GST-RadA_35-252_ (100 nM) and subjected to solid-phase binding assay, as described above.

### 4.8. Binding Assay Using Human Intestinal Epithelial Cells

To study the interaction of GST-RadA_35-252_ with human intestinal epithelial cells, Caco-2 and HT29-16E cells were used as models of human enterocytes and goblet cells, respectively. Caco-2 (ATCC HTB-37) and HT29-16E cells (a generous gift from Prof. Christian Laboisse [40,41] were routinely grown onto 25 cm^2^ flasks in DMEM supplemented with 10% fetal calf serum, 1% L-glutamine, and 1% antibiotics (Thermo Fisher Scientific) and maintained in a 5% CO_2_ incubator at 37 °C. Cells were detached from 25 cm^2^ flasks using trypsin-EDTA solution (Thermo Fisher Scientific), counted using Malassez counting chamber, diluted in culture media, seeded into 96-well cell culture plates (Greiner Bio-one, Paris, France) at approximately 10^4^ cells per well and let to differentiate for 10–14 days [42]. The interaction of GST-RadA_35-252_ with human cells was measured using cell-based ELISA assay as previously described [43]. Briefly, cells were washed once with PBS and fixed with 4% paraformaldehyde (PFA) for 20 min at room temperature. After fixation, wells were washed three times with PBS and were saturated for 1 h at 37 °C with 230 µL of 2% (*w*/*v*) gelatin dissolved in PBS. Wells were then incubated at 37 °C for 2 h with increasing concentrations of GST-RadA_35-252_ (from 0 to 400 nM) diluted in PBS + gelatin 2%. After three washes with PBS, GST-RadA_35-252_ bound to the cells was detected by incubation for 1 h at 37 °C with rabbit anti-GST antibody (1:400 dilution) (Thermo Fisher Scientific). Pure GST (Merck, Fontenay-sous-Bois, France) was used as negative control and showed no interaction with Caco-2 or HT29-16E cells (data not shown). After three washes with PBS, peroxidase-conjugated goat anti-rabbit antibody (1:10,000 dilution) (Jackson ImmunoResearch Laboratories, Cambridgeshire, United Kingdom) was added to the wells and incubated 1 h at 37 °C. Wells were then washed six times with PBS and incubated with 100 µL of the peroxidase substrate o-phenylendiamine dihydrochloride (Sigma-Aldrich, Lyon, France) for 30 min at room temperature before the reaction was stopped by adding 50 µL of H_2_SO_4_. Optical density (OD) at 490 nm was measured using a microplate reader (Biotek, Synergy Mx, Colmar, France). The interaction of GST-RadA_35-252_ (100 nM) with the human intestinal cells was also measured after 30 min pre-incubation of the cells at 37 °C with lectins (Wheat Germ Agglutinin (WGA), Ulex Europaeus Agglutinin (UEA) and Jacalin (JAC) (Vector Laboratories, Peterborough, UK) diluted at 100 µg/mL or after 30 min pre-incubation at 37 °C of GST-RadA_35-252_ (100 nM) with 500 mM of Gal or GalNAc.

### 4.9. Statistical Analysis

All experiments were conducted in triplicate. Graphs were drawn and fitted using GraphPad^®^ Prism 7 software. Binding parameters (i.e., binding capacity (Bmax) and affinity constant (Km)) were determined also using GraphPad^®^ Prism 7 software. *t*-test and two-way ANOVA analyses were used to address the significant differences between mean values, with significance set at *p* < 0.05.

## Figures and Tables

**Table 1:** Binding parameters of GST-RadA_35-252_ on different coated proteins. Data shown in Figure 4 were analyzed using GraphPad software. Collagens, immunoglobulins, mucins and gelatin were coated at 4 pmol per wells (40 nM). Interactions of GST-RadA_35-252_ was measured in term of B_max_ (binding capacity, i.e., maximal OD observed) and Km (affinity constant in nM). Results are expressed as means ± S.D. (*n* = 3).

Coated protein	B_max_	Km (nM)
**Collagen I**	0.82 ± 0.11	161.9 ± 23.81
**Collagen IV**	1.16 ± 0.14	112.5 ± 21.86
**Human IgG**	3.92 ± 0.47	13.75 ± 0.34
**Human IgA**	2.14 ± 0.46	24.71 ± 1.86
**Goat IgG**	1.05 ± 0.06	58.89 ± 9.90
**Rabbit IgG**	0.69 ± 0.32	34.22 ± 5.61
**Mouse IgG**	0.38 ± 0.11	39.82 ± 7.21
**Mucin type I**	0.08 ± 0.02	110.1 ± 30.12
**Mucin type II**	2.56 ± 0.23	16.61 ± 0.46
**Mucin type III**	1.06 ± 0.38	78.52 ± 6.43
**Gelatin**	0.38 ± 0.13	66.18 ± 18.49

**Table 2:** Inhibition of the interaction of GST-RadA_35-252_ with type II mucin by monosaccharides in solution. GST-RadA_35-252_ was incubated with monosaccharides in solution before being added to coated type II mucin as explained in Materials and Methods. Tested monosaccharides were Fructose (Frc), Fucose (Fuc), Galactose (Gal), N-acetylgalactosamine (GalNAc), Glucose (Glc), N-acetylglucosamine (GlcNAc), Mannose (Man) and Sialic acid. IC_50_ values (i.e., the concentration of monosaccharide inhibiting 50% of the interaction of GST-RadA_35-252_ with type II mucin) were calculated from Figure 6 using GraphPad Prism. Results are expressed as means ± S.D. (*n* = 3).

Monosaccharide	IC_50_ (mM)	% of Inhibition at 500 mM
Frc	23.0 ± 8.44	1.61 ± 2.18
Fuc	29.04 ± 8.36	2.73 ± 2.42
Gal	937.01 ± 173.38	34.68 ± 2.73
GalNAc	506.12 ± 139.19	51.61 ± 3.41
Glc	5.28 ± 1.06	10.75 ± 6.38
GlcNAc	25.98 ± 4.52	2.18 ± 4.36
Man	14.00 ± 2.89	2.82 ± 1.62
Sialic acid	1685.32 ± 576.84	17.94 ± 5.81

**Table 3:** Primers sequences. PCR was performed in a 50 µL-final volume reaction mixture containing 1 µM of each primer, 250 µM of each dNTP, 1.5–2.5 mM of MgCl2 and DNA template, with a Mastercycler Gradient (Eppendorf). Primers Ol35-Eco and Ol252-Xho were used to amplify the DNA fragment encoding the RadA region spanning from L35 to L252. They harbored an *Eco*RI or *Xho*I restriction site, respectively, for further cloning. The PCR program used was as follows: (1) initial denaturation at 95 °C for 5 min, (2) denaturation at 95 °C for 40 s, (3) annealing for 40 s at 55 °C, (4) extension at 72 °C for 40 s, and (5) a final extension at 72 °C for 10 min, with the second to fourth steps repeated for 24 cycles. Primers C1L, C1R, C3L, C3R, C5L, C5R, C6L and C6R were used in PCR reactions carried out on R. gnavus E1 cDNA obtained from cells colonizing the caecum of mono-contaminated animals (in situ cDNA) or grown in BHI-YH medium (in vivo DNA). Control experiments were carried out on E1 chromosome DNA. The PCR program used was as follows: (1) initial denaturation at 95 °C for 5 min, (2) denaturation at 95 °C for 40 s, (3) annealing for 40 s at 58 °C, (4) extension at 72 °C for 40 s, and (5) a final extension at 72 °C for 10 min, with the second to fourth steps repeated for 29 cycles.

Primer	Sequence
Ol35-Eco	5′-CCGGAATTCTTAGAACAGTCAGAGAATAAAGCG-3′
Ol252-Xho	5′-CCGCTCGAGATTTAAATATTCTCCGGTAAGATCACCCGG-3′
Ad1F	5′-CGGCTTCTGATTTTAAAGGGATTAC-3′
Ad2R	5′-GTTTTGCACTTGGCTCTTCA-3′
Ad2F	5′-GAAGAGCCAAGTGCAAAAG-3′
Ad3R	5′-CTGAAAGGTGTGTGTAAAAGTG-3′
Ad3F	5′-CACTTTTACACACACCTTTCAG-3′
Ad4R	5′-GTCACCTCATTTAATGGAAG-3′
Ad5F	5′-CATGAGGAACAGGCTCCAAT-3′
Ad6R	5′-TCTTTGTGCGTCTGATTCTC-3′

## References

[1] Bry L., Falk P.G., Midtvedt T., Gordon J.I. (1996). A Model of Host-Microbial Interactions in an Open Mammalian Ecosystem. Science.

[2] Hooper L.V., Xu J., Falk P.G., Midtvedt T., Gordon J.I. (1999). A Molecular Sensor That Allows a Gut Commensal to Control Its Nutrient Foundation in a Competitive Ecosystem. Proc. Natl. Acad. Sci. USA.

[3] Pizarro-Cerdá J., Cossart P. (2006). Bacterial Adhesion and Entry into Host Cells. Cell.

[4] Stones D.H., Krachler A.M. (2016). Against the Tide: The Role of Bacterial Adhesion in Host Colonization. Biochem. Soc. Trans..

[5] Susmitha A., Bajaj H., Madhavan Nampoothiri K. (2021). The Divergent Roles of Sortase in the Biology of Gram-Positive Bacteria. Cell Surf..

[6] Madani A., Garakani K., Mofrad M.R.K. (2017). Molecular Mechanics of Staphylococcus Aureus Adhesin, CNA, and the Inhibition of Bacterial Adhesion by Stretching Collagen. PLoS ONE.

[7] Rich R.L., Kreikemeyer B., Owens R.T., LaBrenz S., Narayana S.V., Weinstock G.M., Murray B.E., Höök M. (1999). Ace Is a Collagen-Binding MSCRAMM from Enterococcus Faecalis. J. Biol. Chem..

[8] Nallapareddy S.R., Weinstock G.M., Murray B.E. (2003). Clinical Isolates of Enterococcus Faecium Exhibit Strain-Specific Collagen Binding Mediated by Acm, a New Member of the MSCRAMM Family. Mol. Microbiol..

[9] Miller J.H., Avilés-Reyes A., Scott-Anne K., Gregoire S., Watson G.E., Sampson E., Progulske-Fox A., Koo H., Bowen W.H., Lemos J.A. (2015). The Collagen Binding Protein Cnm Contributes to Oral Colonization and Cariogenicity of Streptococcus Mutans OMZ175. Infect. Immun..

[10] Liu Q., Ponnuraj K., Xu Y., Ganesh V.K., Sillanpää J., Murray B.E., Narayana S.V.L., Höök M. (2007). The Enterococcus Faecalis MSCRAMM ACE Binds Its Ligand by the Collagen Hug Model. J. Biol. Chem..

[11] Johansson M.E.V., Ambort D., Pelaseyed T., Schütte A., Gustafsson J.K., Ermund A., Subramani D.B., Holmén-Larsson J.M., Thomsson K.A., Bergström J.H. (2011). Composition and Functional Role of the Mucus Layers in the Intestine. Cell. Mol. Life Sci..

[12] Cantarel B.L., Coutinho P.M., Rancurel C., Bernard T., Lombard V., Henrissat B. (2009). The Carbohydrate-Active EnZymes Database (CAZy): An Expert Resource for Glycogenomics. Nucleic Acids Res..

[13] Flint H.J., Scott K.P., Duncan S.H., Louis P., Forano E. (2012). Microbial Degradation of Complex Carbohydrates in the Gut. Gut Microbes.

[14] Marcobal A., Southwick A.M., Earle K.A., Sonnenburg J.L. (2013). A Refined Palate: Bacterial Consumption of Host Glycans in the Gut. Glycobiology.

[15] Jensen P.H., Kolarich D., Packer N.H. (2010). Mucin-Type O-Glycosylation--Putting the Pieces Together. FEBS J..

[16] Moran A.P., Gupta A., Joshi L. (2011). Sweet-Talk: Role of Host Glycosylation in Bacterial Pathogenesis of the Gastrointestinal Tract. Gut.

[17] Juge N. (2012). Microbial Adhesins to Gastrointestinal Mucus. Trends Microbiol..

[18] Roos S., Jonsson H. (2002). A High-Molecular-Mass Cell-Surface Protein from Lactobacillus Reuteri 1063 Adheres to Mucus Components. Microbiology.

[19] MacKenzie D.A., Tailford L.E., Hemmings A.M., Juge N. (2009). Crystal Structure of a Mucus-Binding Protein Repeat Reveals an Unexpected Functional Immunoglobulin Binding Activity. J. Biol. Chem..

[20] Qin J., Li R., Raes J., Arumugam M., Burgdorf K.S., Manichanh C., Nielsen T., Pons N., Levenez F., Yamada T. (2010). A Human Gut Microbial Gene Catalogue Established by Metagenomic Sequencing. Nature.

[21] Ramare F., Nicoli J., Dabard J., Corring T., Ladire M., Gueugneau A.M., Raibaud P. (1993). Trypsin-Dependent Production of an Antibacterial Substance by a Human Peptostreptococcus Strain in Gnotobiotic Rats and in Vitro. Appl. Environ. Microbiol..

[22] Gomez A., Ladiré M., Marcille F., Fons M. (2002). Trypsin Mediates Growth Phase-Dependent Transcriptional Tegulation of Genes Involved in Biosynthesis of Ruminococcin A, a Lantibiotic Produced by a Ruminococcus Gnavus Strain from a Human Intestinal Microbiota. J. Bacteriol..

[23] Crost E.H., Ajandouz E.H., Villard C., Geraert P.A., Puigserver A., Fons M. (2011). Ruminococcin C, a New Anti-Clostridium Perfringens Bacteriocin Produced in the Gut by the Commensal Bacterium Ruminococcus Gnavus E1. Biochimie.

[24] Midtvedt T. (1986). Effects of Antimicrobial Agents upon the Functional Part of the Intestinal Flora. Scand. J. Infect. Dis. Suppl..

[25] Chiumento S., Roblin C., Kieffer-Jaquinod S., Tachon S., Leprètre C., Basset C., Aditiyarini D., Olleik H., Nicoletti C., Bornet O. (2019). Ruminococcin C, a Promising Antibiotic Produced by a Human Gut Symbiont. Sci. Adv..

[26] Roblin C., Chiumento S., Bornet O., Nouailler M., Müller C.S., Jeannot K., Basset C., Kieffer-Jaquinod S., Couté Y., Torelli S. (2020). The Unusual Structure of Ruminococcin C1 Antimicrobial Peptide Confers Clinical Properties. Proc. Natl. Acad. Sci. USA.

[27] Graziani F., Pujol A., Nicoletti C., Dou S., Maresca M., Giardina T., Fons M., Perrier J. (2016). Ruminococcus Gnavus E1 Modulates Mucin Expression and Intestinal Glycosylation. J. Appl. Microbiol..

[28] Pujol A., Crost E.H., Simon G., Barbe V., Vallenet D., Gomez A., Fons M. (2011). Characterization and Distribution of the Gene Cluster Encoding RumC, an Anti-Clostridium Perfringens Bacteriocin Produced in the Gut. FEMS Microbiol. Ecol..

[29] Zong Y., Xu Y., Liang X., Keene D.R., Höök A., Gurusiddappa S., Höök M., Narayana S.V.L. (2005). A “Collagen Hug” Model for Staphylococcus Aureus CNA Binding to Collagen. EMBO J..

[30] Chagnot C., Listrat A., Astruc T., Desvaux M. (2012). Bacterial Adhesion to Animal Tissues: Protein Determinants for Recognition of Extracellular Matrix Components. Cell. Microbiol..

[31] Mack D.R., Ahrne S., Hyde L., Wei S., Hollingsworth M.A. (2003). Extracellular MUC3 Mucin Secretion Follows Adherence of Lactobacillus Strains to Intestinal Epithelial Cells in Vitro. Gut.

[32] Deivanayagam C.C., Rich R.L., Carson M., Owens R.T., Danthuluri S., Bice T., Höök M., Narayana S.V. (2000). Novel Fold and Assembly of the Repetitive B Region of the *Staphylococcus Aureus* Collagen-Binding Surface Protein. Structure.

[33] Miljkovic M., Bertani I., Fira D., Jovcic B., Novovic K., Venturi V., Kojic M. (2016). Shortening of the *Paracasei Subsp. Paracasei* BGNJ1-64 AggLb Protein Switches Its Activity from Auto-Aggregation to Biofilm Formation. Front. Microbiol..

[34] Crost E.H., Tailford L.E., Le Gall G., Fons M., Henrissat B., Juge N. (2013). Utilisation of Mucin Glycans by the Human Gut Symbiont Ruminococcus Gnavus Is Strain-Dependent. PLoS ONE.

[35] Bruel L., Sulzenbacher G., Cervera Tison M., Pujol A., Nicoletti C., Perrier J., Galinier A., Ropartz D., Fons M., Pompeo F. (2011). α-Galactosidase/Sucrose Kinase (AgaSK), a Novel Bifunctional Enzyme from the Human Microbiome Coupling Galactosidase and Kinase Activities. J. Biol. Chem..

[36] Aguilera M., Rakotoarivonina H., Brutus A., Giardina T., Simon G., Fons M. (2012). Aga1, the First Alpha-Galactosidase from the Human Bacteria Ruminococcus Gnavus E1, Efficiently Transcribed in Gut Conditions. Res. Microbiol..

[37] Cervera-Tison M., Tailford L.E., Fuell C., Bruel L., Sulzenbacher G., Henrissat B., Berrin J.G., Fons M., Giardina T., Juge N. (2012). Functional Analysis of Family GH36 α-Galactosidases from Ruminococcus Gnavus E1: Insights into the Metabolism of a Plant Oligosaccharide by a Human Gut Symbiont. Appl. Environ. Microbiol..

[38] Dabard J., Bridonneau C., Phillipe C., Anglade P., Molle D., Nardi M., Ladiré M., Girardin H., Marcille F., Gomez A. (2001). Ruminococcin A, a New Lantibiotic Produced by a Ruminococcus Gnavus Strain Isolated from Human Feces. Appl. Environ. Microbiol..

[39] Vallenet D., Calteau A., Dubois M., Amours P., Bazin A., Beuvin M., Burlot L., Bussell X., Fouteau S., Gautreau G. (2020). MicroScope: An Integrated Platform for the Annotation and Exploration of Microbial Gene Functions through Genomic, Pangenomic and Metabolic Comparative Analysis. Nucleic Acids Res..

[40] Augeron C., Laboisse C.L. (1984). Emergence of Permanently Differentiated Cell Clones in a Human Colonic Cancer Cell Line in Culture after Treatment with Sodium Butyrate. Cancer Res..

[41] Graziani F., Pinton P., Olleik H., Pujol A., Nicoletti C., Sicre M., Quinson N., Ajandouz E.H., Perrier J., Pasquale E.D. (2019). Deoxynivalenol Inhibits the Expression of Trefoil Factors (TFF) by Intestinal Human and Porcine Goblet Cells. Arch. Toxicol..

[42] Ajandouz E.H., Berdah S., Moutardier V., Bege T., Birnbaum D.J., Perrier J., Di Pasquale E., Maresca M. (2016). Hydrolytic Fate of 3/15-Acetyldeoxynivalenol in Humans: Specific Deacetylation by the Small Intestine and Liver Revealed Using in Vitro and Ex Vivo Approaches. Toxins.

[43] Razafimanjato H., Benzaria A., Taïeb N., Guo X.-J., Vidal N., Di Scala C., Varini K., Maresca M. (2011). The Ribotoxin Deoxynivalenol Affects the Viability and Functions of Glial Cells. Glia.

